# Image analysis-based discoloration rate quantification and kinetic modeling for shelf-life prediction in herb-coated pear slices

**DOI:** 10.1038/s41598-024-51840-y

**Published:** 2024-01-18

**Authors:** Sathya R., Prasad Rasane, Aishvina Singh, Jyoti Singh, Sawinder Kaur, Vikas Nanda, Jaspreet Kaur, Mahendra Gunjal, Vishesh Bhadariya, Sezai Ercisli, Riaz Ullah, Essam A. Ali

**Affiliations:** 1 Department of Food Technology and Nutrition, School of Agriculture, Lovely Professional University, Phagwara, Punjab 144411 India; 2 Sant Longowal Institute of Engineering and Technology, Sangrur, Punjab 148106 India; 3 School of Chemical Engineering, Oklahoma State University, Stillwater, OK-74078 USA; 4 Department of Horticulture, Faculty of Agriculture, Ataturk University, 25240 Erzurum, Turkey; 5 HGF Agro, ATA Teknokent, TR-25240 Erzurum, Turkey; 6 Department of Pharmacognosy, College of Pharmacy, King Saud University, Riyadh, Saudi Arabia; 7 Department of Pharmaceutical Chemistry, College of Pharmacy, King Saud University, Riyadh, Saudi Arabia

**Keywords:** Biochemistry, Biological techniques

## Abstract

The present research study aimed to examine three different herb extract's effects on the discoloration rate of fresh-cut pear slices using an image analysis technique. Pear slices were sprayed and dip-coated with *Ocimum basilicum, Origanum vulgare,* and *Camellia sinensis* (0.1 g/ml) extract solution. During 15 days storage period with three days intervals, all sprayed/dip-coated pear slices were analyzed for the quality attribute (TA) and color parameters notably a*, b*, hue angle (H*), lightness (L*), and total color change (ΔE). Further, order kinetic models were used to observe the color changes and to predict the shelf-life. The results obtained showed that the applicability of image analysis helped to predict the discoloration rate, and it was better fitted to the first-order (FO) kinetic model (R^2^ ranging from 0.87 to 0.99). Based on the kinetic model, color features ΔE and L* was used to predict the shelf-life as they had high regression coefficient values. Thus, the findings obtained from the kinetic study demonstrated *Camellia sinensis (assamica)* extract spray-coated pear slices reported approximately 28.63- and 27.95-days shelf-stability without much discoloration compared with all other types of surface coating.

## Introduction

Pear (*Pyrus communis* ‘Comice’) is a fruit belonging to the genus Pyrus and family Rosaceae, characterized by hard-seeded fleshy berries with thick fruit peel^1^. The size, color, and shape of pears can vary between individual fruits. Pears have a high sugar content and low acidity, resulting in a sweeter and more delicious taste in comparison with apples. Melatonin, a compound found in pears, can increase fruit size by enhancing the net photosynthetic rate and increasing soluble sugar content^2^. Pears are also a good source of fiber, vitamin C, and minerals such as potassium, sodium, calcium, magnesium, and iron^3,4^.

Regular consumption of pears has been associated with various health benefits. They can help alleviate intestinal inflammation, constipation, and kidney stones, and are included in diets due to their low caloric content^5^. However, pears are susceptible to enzymatic reactions, leading to discoloration and limited shelf life in their fresh state^6^.

To meet the increasing market demand for cut pear slices, it is important to prevent rapid discoloration and maintain freshness over an extended period. Chemical anti-browning agents, particularly sulfites, have been used to prevent surface browning. However, these agents are not considered safe for regular consumption and can cause adverse effects such as dizziness, nausea, and potentially life-threatening diseases^7^. Additionally, they may contribute to nutrient loss and reduce the overall health benefits of the fruit.

As an alternative, researchers and food processors have turned to edible coatings made from natural herb, vegetable, or fruit extracts, particularly herbs with antioxidant properties. Medicinal herbs contain high levels of natural phenolic compounds, including phenolic acids, flavonoids, quinones, lignans, tannins, and stilbenes, which possess antioxidant and free-radical scavenging properties^8–11^. Thus in this study herbs such as *Camellia sinensis assamica* (Green tea), *Origanum vulgare* (Oregano) and *Ocimum basilicum* (Basil) were chosen as the coating material.

*Camellia sinensis assamica* (Green tea) is a member of the Theaceae family contains effective polyphenolic structures such as catechins, flavones, phenolic acids, and anthocyanins, which have potent antioxidant properties^12–14^. *Origanum vulgare* (Oregano) from the Lamiaceae family contains various phenolic compounds, including phenolic acids, hydroxycinnamic acids, stilbenes, and flavonoids, which contribute to its antioxidant activity^15,16^. *Ocimum basilicum* (Basil) also belongs to the Lamiaceae family containing phenolic compounds such as hydroxybenzoic acid, flavonol glycosides, and phenolic acids, which have antioxidant and anti-browning properties^17,18^.Thus, all the three selected herbs due to its effective polyphenolic structures has potent antioxidant properties that linearly helps to reduce the enzyme activity.

In a recent research study, dried leaf extracts of *Camellia sinensis*, *Origanum vulgare*, and *Ocimum basilicum* were coated onto pear slices at a concentration of 0.1 g/ml. The coated pear slices were then analyzed using image analysis software to determine the rate of discoloration based on total color change (ΔE) during storage. Image analysis software allows for accurate and area-specific measurement of discoloration rates, eliminating the subjectivity of manual inspection and reducing labor costs^19^. Various properties such as color, frequency domain measures, statistical greatness, and pixel distribution values were extracted from the images using this software^20^.

Image analysis has been successfully applied in similar studies, such as determining the color and shelf-life prediction of apple pomace with wine^21^ and assessing browning inhibition rates in fresh-cut apples using a computer vision system^22^. These studies have demonstrated the increased accuracy of assessment provided by image analysis techniques. Therefore, the present research aims to analyze the appearance of *Camellia sinensis*, *Ocimum basilicum*, and *Origanum vulgare*-coated pear slices during a 15-day storage period at 4 °C using image analysis techniques and kinetics-based shelf-life testing (SLT) approaches, focusing on lightness (L*) and total color change (ΔE).

## Result and discussion

### Coating effect on titratable acidity

**Figure 1:**
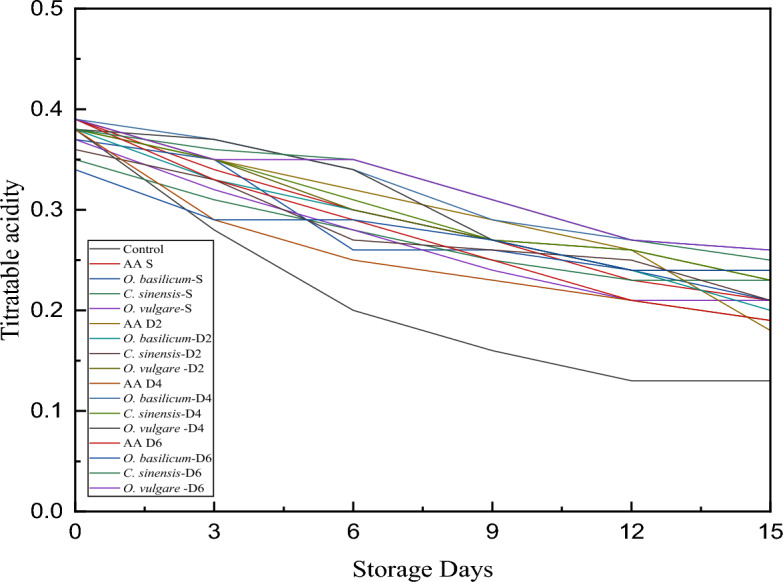
Effect of coating on titratable acidity of fresh-cut pear slices under cold storage (4 ± 1 °C ). AA S—Ascorbic Acid Spray; *O. basilicum* S—*O. basilicum* Spray; *C. sinensis* S—C. sinensis S—Spray; *O. vulgare* S—*O. vulgare* Spray; *O. basilicum* D2—O. basilicum Dip 2 min; *C. sinensis* S—*C. sinensis* D2—2 min; *O. vulgare* D2—*O. vulgare* Dip 2 min; *O. basilicum* D4—*O. basilicum* Dip 4 min; *C. sinensis* S—*C. sinensis* D4—4 min; *O. vulgare* D4—*O. vulgare* Dip 4 min; *O. basilicum* D6—*O. basilicum* Dip 6 min; *C. sinensis* S—*C. sinensis* D6—6 min; *O. vulgare* D6—*O. vulgare* Dip 6 min.

Figure 1 represents that the titratable acidity of the pear slices decreased during 15 days of storage at 4 °C. Titratable acidity decline was highest in the case of control pear slices which ranged from 0.38 ± 0.01 at 0 days to 0.13 ± 0.03 at 15th day of storage. A comparative study of two coating techniques denoted dip coating for 6 min retained more acid content and the decrease was slow compared to spray-coated pear slices. The acid content reduction was lowest in the case of *C. sinensis* dip-coated pear slices, it showed a minimum fall in the acid content (0.39 ± 0.03 to 0.26 ± 0.26) followed by *O. vulgare* dip-coated pear slices (0.39 ± 0.01 to 0.26 ± 0.14). The overall acid content fall was reported in the sequence of *O. basilicum C. sinensis* > *O. vulgare* > *C. sinensis*. The decline in the acid content during the storage period is due to the utilization of organic acid (citrus/malic acid) in the metabolic and respiration processes of the pear slices^23^.

As the fruit ripens, the titratable acidity of the sample gradually decreases. A study by Mani et al.^23^ in aloe vera edible coated ber fruit reported a decreased acid content of coated and uncoated ber fruit, whereby the acid content decline of uncoated ber fruit was more over coated ber fruit with the passage of the storage period at 4 °C. Thus, supporting these results, our study also suggests that the titratable acidity in the uncoated pear slices were significantly lower as compared to that of herb extract coated pear slices. The results from this examination are in agreement with the study by Zheng et al.^24^ who treated pear slices with melatonin, to inhibit enzymatic browning and preserve the quality attributes, this study suggested that melatonin coating to the fruit samples could effectively reduce the transpiration rate, this may be due to retarred respiration rate which is a consequence of declined organic acid content. The findings of mentioned research were reviewed to support the current study. Thus, a decline in the titratable acidity during the storage period makes the herbs-coated pear slices lesser acidic whereby it can be best suitable for consumption due to taste.

### Changes in the color value of coated pear slices using image analysis

**Figure 2:**
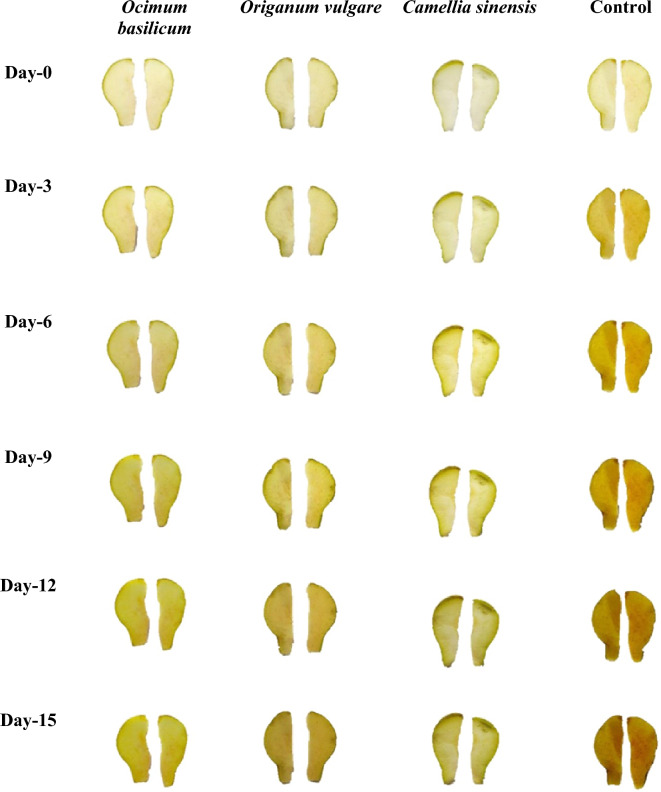
*Ocimum basilicum, Origanum vulgare* and *Camellia sinensis* extract spray coated pear slices captured over 15 days storage.

**Figure 3:**
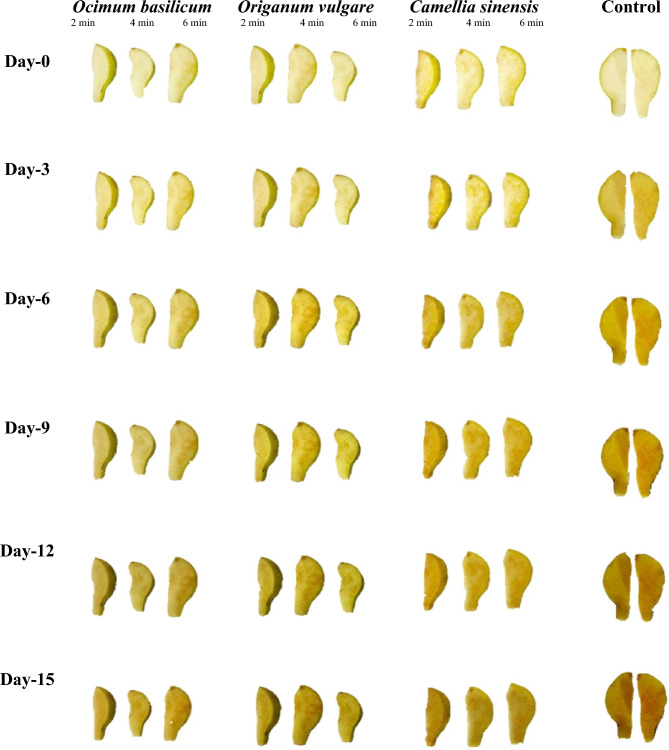
*Ocimum basilicum, Origanum vulgare* and *Camellia sinensis* extract dip (2, 4, 6 min) coated pear slices captured over 15 days storage.

**Figure 4:**
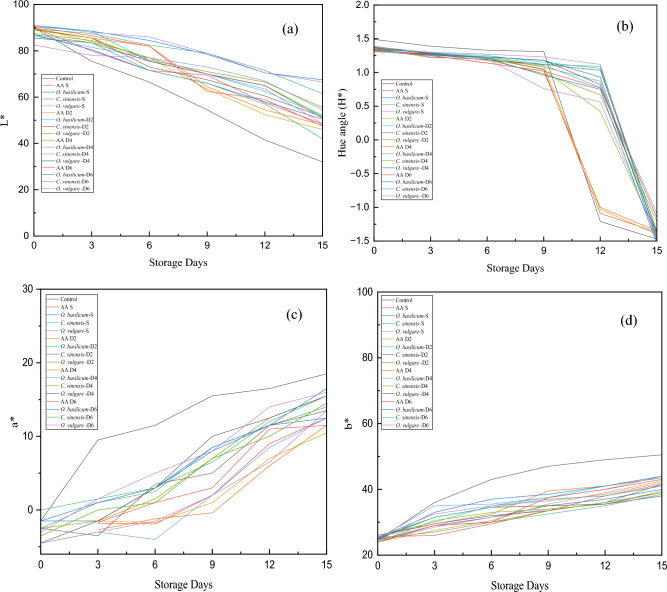
Effect of (**a**) redness (a*), (**b**) yellowness (b*), (**c**) lightness (L*), (**d**) Hue angle (H*) of fresh-cut pear slices under cold storage (4 ± 1 °C). AAS, Ascorbic Acid Spray; *O. basilicum* S—*O. basilicum* Spray; *C. sinensis* S—C. sinensis S, Spray; *O. vulgare* S—*O. vulgare* Spray; *O. basilicum* D2—O. basilicum Dip 2 min; *C. sinensis* S—*C. sinensis* D2—2 min; *O. vulgare* D2—*O. vulgare* Dip 2 min; *O. basilicum* D4—*O. basilicum* Dip 4 min; *C. sinensis* S—*C. sinensis* D4—4 min; *O. vulgare* D4—*O. vulgare* Dip 4 min; *O. basilicum* D6—*O. basilicum* Dip 6 min; *C. sinensis* S—*C. sinensis* D6—6 min; *O. vulgare* D6—*O. vulgare* Dip 6 min.

The image captured from pear slices were processed during the experiments on days 0, 3, 6, 9, 12, and 15. As the color features and parameters change during storage days, the frequency of the extracted color parameters (RGB) also change for each coated and uncoated pear slice concerning time. These three features (RGB) are suitable for determining the discoloration rate of pear slices. Figures 2, and 3 show the browning process of the spray/dip-coated pear slices during the storage period. As indicated in Fig. 2 and 3, it is noticed that slowly along with the increase in the discoloration/dark pigment formation on the surface of pear slices, RGB color parameter distribution is also changed. Using image analysis software “Adobe Photoshop (CS3 extended)” the color parameter RGB value is converted to LAB color scale. LAB values extracted from the captured image showed a decrease in the value of L* and H* as indicated in Fig. 4a,b during 15 days of storage. The final reduction in the value of L* for *O. basilicum, O. vulgare* and *C. sinensis* spray-coated pear slices ranged from 91 ± 0.01 to 67.5 ± 0.76, in the case of *O. basilicum, O. vulgare,* and *C. sinensis* dip coated (2 min, 4 min, 6 min) pear slices ranged from 90.5 ± 0.70 to 52 ± 0.11, 85.5 ± 1.12 to 51 ± 0.21, 87 ± 2.13 to 42 ± 0.01, respectively. These findings clearly showed dip coated (6 min) and control pear slices gradually increased the browning rate, wherein control samples accelerated browning due to the activity of phenolase and peroxidase. The same trend was observed by Subhashree et al.^25^ in apples, the images in this study were captured every 5 min for a total period of 14 h, and comparative analysis of 168 images indicated a decrease in lightness value for 14 h. Thus, as the storage time increases the discoloration rate of samples also gradually increases.

The variation in redness (a*) during 15 days of storage is shown in Fig. 4c. An increase from − 1.5 to 17, − 2.5 to 13, and − 4.5 to 15.5 in the values of a* was observed in *O. basilicum, O. vulgare,* and *C. sinensis* spray-coated pear slices. Whereby, an increase from − 3.5 to 14.5, − 3.5 to 14.5, and − 3 to 16.1 in the values of a* was observed in *O. basilicum, O. vulgare,* and *C. sinensis* dip (6 min) coated pear slices. It was noticed that the extension of storage days simultaneously changed the original color of pear slices to a brownish shade after 3 days, and in the case of control samples, they changed their original color to a brownish shade within a day. The finding of the study investigated by Htike et al.^26^ in guava using image processing techniques, showed similar results that supported our study, this study in guava fruit using captured images (ROI) showed a decrease in the lightness value and an increase in a* value correlatively indicate sample’s discoloration rate.

During the storage period these coated pear slices slowly start to degrade and lose their yellow color, thus, b* values gradually increase as described in Fig. 4d, this increased value of b* determines that the *O. basilicum, O. vulgare* and *C. sinensis* spray/dip-coated pear slices induce luminosity loss and changed to a darker color. Based on the current study, it was revealed that *O. basilicum* spray-coated pear slices obtained b* values ranging from 24.5 to 40, *O. vulgare* spray-coated pear slices ranged from 24 to 39, *C. sinensis* spray-coated pear slices ranged from 25 to 38.5. The overall loss of luminosity (b*) in spray-coated pear slices ranges in the sequence of *O. vulgare* > *O. basilicum* > *C. sinensis*. A similar trend was observed in a study conducted by Wong et al.^27^, in which apple, potato, and pineapple were dipped (5 min) in extracts of *Centella asiatica* and stored at 1 °C, this study supported our work whereby denoting decrease in the lightness value during 4 h storage and loss of luminosity/increase in b* value correlatively increased total color difference and the study also indicated higher the L* value lower the browning index value. Thus, the overall increase in the value of b* shown in fresh cut apple is due to the partial formation of discoloration pigments like melanins/quinones^22^.

A homogenous rate of discoloration was observed in coated pear slices. Compared with food colorimeter or hunter lab colorimeter measurement, this image analysis software helps in breaking down the complete surface of the captured images in RGB value and this RGB value can be converted to LAB value, that further can be utilized for the determination of discoloration rate. Rana et al.^28^ and Shomodder et al.^29^ reported that in fruits the L* value decreased and the a* values increased during browing stage.

### Relationship between total color difference and storage of coated pear slices

**Figure 5:**
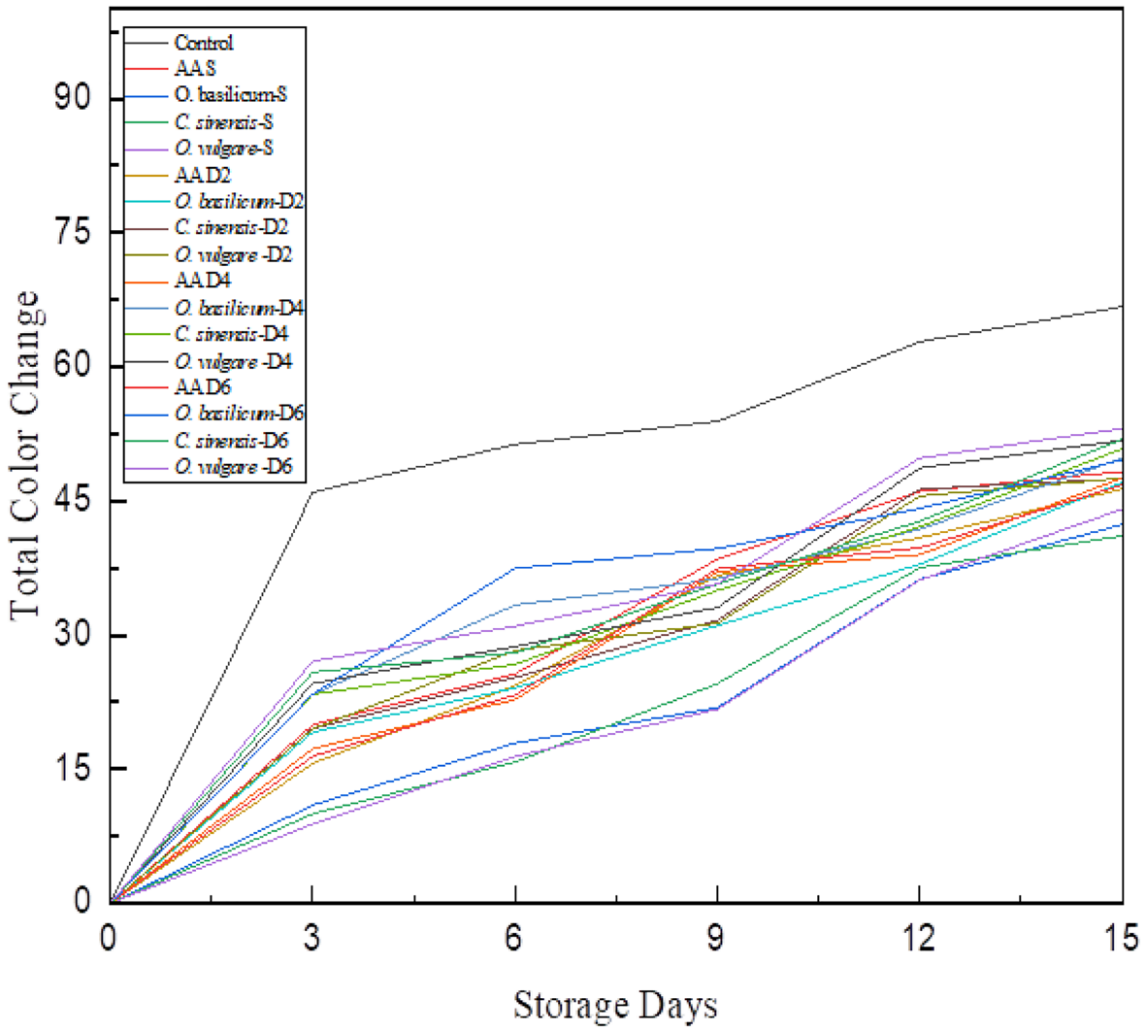
Effect of coating on total color change of fresh-cut pear slices under cold storage (4 ± 1 °C) AAS—Ascorbic Acid Spray; *O. basilicum* S—*O. basilicum* Spray; *C. sinensis* S—C. sinensis S—Spray; *O. vulgare* S—*O. vulgare* Spray; *O. basilicum* D2—O. basilicum Dip 2 min; *C. sinensis* S—*C. sinensis* D2—2 min; *O. vulgare* D2—*O. vulgare* Dip 2 min; *O. basilicum* D4—*O. basilicum* Dip 4 min; *C. sinensis* S—*C. sinensis* D4—4 min; *O. vulgare* D4—*O. vulgare* Dip 4 min; *O. basilicum* D6—*O. basilicum* Dip 6 min; *C. sinensis* S—*C. sinensis* D6—6 min; *O. vulgare* D6—*O. vulgare* Dip 6 min.

Total Color Difference (ΔE) was determined to estimate the discoloration rate in fresh-cut pear slices. ΔE of control pear slices was high after three days of storage (at a level of statistical significance *p* < 0.01). Phenolase (EC 1.10.3.1) and peroxidase (EC 1.11.1.7) are discoloration enzymes that are amenable to elevate the total color difference, and the activity of these enzymes is accelerated during storage days^24^. As described in Fig. 5, the discoloration rate during day 0 and day 3 was mildest and in correlation to these results, it can be observed that discoloration enzyme activity from day 0 to day 3 was less in comparison to day 6 to day 15 due to effectiveness of herb-coating that act as a barrier preventing oxygen interacting with fruit tissues. In the case of control pear slices, the discoloration rate rapidly elevated as no herb-coating was applied to the control pear slices. Thus, the signs of complete discoloration/browning were also noticeable in control pear slices due the enzymatic action of polyphenol oxidase. From the results procured from image analysis, it is clear control samples developed browning more quickly in comparison with herb-coated pear slices.

Whereas in the case of *O. basilicum*, *O. vulgare,* and *C. sinensis* extract-coated pear slices the ΔE was low until day 9. The value of ΔE for *O. basilicum* extract spray-coated pear slices on day 9 was 21.9 which suddenly elevated to 36.23 on day 12, *O. vulgare* extract spray-coated pear slices on day 9 was 24.33 which elevated to 37.53 on day 12, similarly *C. sinensis* extract spray coated pear slices on day 9 was 21.69 which suddenly elevated to 36.16 on day 12. All the three-extract coated in the form of spraying showed beneficial results, and the overall ΔE was reported to increase in the sequence of *O. vulgare* > *O. basilicum* > *C. sinensis*. Furthermore, *C. sinensis* spray-coated pear slices showed lower ΔE in comparison with the other dip coating and control samples. Amiri et al.^30^ in their findings concluded *Camellia sinensis* (Green tea) incorporation with aloe vera increased the lightness and overall ΔE was reduced as green tea control oxidative discoloration due to its strong polymeric matrix interaction and polyphenolic compounds, the finding of this research positively supported our results.

Whereas in the case of dip coating (2, 4, and 6 min) pear slices with *O. basilicum*, *O. vulgare,* and *C. sinensis* extract reported similar results as spray coating, but dip coating of 4 and 6 min was not much beneficial as it retained the color (brown) of the extracts, thus the appearance of pear slices showed some discoloration immediately after straining the dip-coated pear slices. The overall ΔE for dip-coated samples was reported to increase in the sequence of *O. vulgare* > *O. basilicum* > *C. sinensis*. Moreover, these findings correlated with previous studies conducted by Wessels et al.^31^ and Salminen et al.^32^ in which *C. sinensis* extract showed the highest anti-browning effect, the myth beyond this method of mechanism is that green tea rich in flavonoid compounds usually have three hydroxyl group in its structure, which thereby induce the activity of tyrosinase (intermediating enzymes for discoloration) by chelating copper, this, in turn, reduce the activity of the PPO as it contains copper in its active site. In some cases, ( +) catechin present in green tea act as a substrate for PPO, on the other hand, esterified catechin, epigallocatechin, and tannin present positively deactivate the PPO activity^33^. Still, there is a contradictory opinion on tannin, as in some cases it inhibits discoloration rate due to its antioxidant/ free radical scavenging properties and in another case, when samples are exposed for more than a minute to extracts containing tannin it imparts its color to sample, thus the mechanism of tannin in inhibition is not elucidated briefly.

### Kinetic analysis in spray/dip-coated pear slices

Order kinetic models were used to describe the changes in the enzymatic browning of the *O. basilicum, O. vulgare,* and *C. sinensis* spray/dip-coated pear slices. Table 1 describes the kinetic parameters obtained from both fittings (zero/first order). The reaction order estimation was achieved from order model fitting (zero/first order) for lightness (L*), total color change (ΔE), and hue angle (H*) of all the coated pear slices. The reaction order of color/quality changes in coated pear slices was determined using R^2^, rate constant (K), and root mean square error (RMSE) as the function of storage days at 4 °C. Based on the model fitting, the lightness and total color difference values of all 3-extract spray/dip coated pear slices fitted better with the first order kinetic model, where R^2^ ranges from 0.87 to 0.99 and K value ranges from 0.0292 ± 0.00341 to 0.2002 ± 0.03654. Meanwhile, the R^2^ value obtained from the zero-order kinetic model ranges from 0.81 to 0.98 and the K value ranges from 1.0618 ± 0.0917 to 4.07251 ± 0.28823. It was observed that R^2^ values were slightly higher for first-order kinetic models compared with zero-order kinetic models. The findings denoted that the changes in lightness and ΔE of coated pear slices for 15 days signified relatively good performance and were well-fitted to first-order kinetics based on the evaluation of rate constant (K), RMSE, and R^2^. Quevedo et al.^34^ compared the effect of the order model and Weibull model to determine the enzymatic browning of pears, mushrooms, avocado, apple, and banana based on lightness and total color difference values obtained during storage period at two temperatures 25 and 35 °C. The study concluded first-order kinetic model showed good performance and fitting but the mean square error (MSE) for the first-order kinetic model was lower compared to the Weibull kinetic model.

**Table 1:** Reaction order estimation for color parameters of Ocimum basilicum, Origanum vulgare and Camellia sinensis extract spray/dip coated pear slices.

Edible coating	Color parameters	Zero order			First order		
		R^2^	K	RMSE	R^2^	K	RMSE
Control	L*	0.89	0.04 ± 0.69	1.10	0.95	0.28 ± 0.06	0.72
	H*	0.72	0.74 ± 1.15	0.91	0.98	0.13 ± 0.04	0.59
	ΔE	0.91	0.86 ± 2.04	0.73	0.93	0.12 ± 0.04	0.34
*O. basilicum*-S	L*	0.82	0.67 ± 0.39	0.86	0.93	0.11 ± 0.08	0.07
	H*	0.98	0.07 ± 0.28	0.19	0.90	0.06 ± 0.02	0.15
	ΔE	0.83	0.51 ± 5.68	0.10	0.99	0.04 ± 0.01	0.04
*C. sinensis*-S	L*	0.97	0.60 ± 0.13	0.20	0.97	0.10 ± 0.01	0.68
	H*	0.92	0.83 ± 0.40	1.10	0.93	0.07 ± 0.03	0.95
	ΔE	0.94	0.50 ± 0.17	0.59	0.92	0.05 ± 0.01	1.09
*O. vulgare*-S	L*	0.92	0.09 ± 0.28	0.13	0.92	0.18 ± 0.03	0.09
	H*	0.98	0.06 ± 0.25	0.99	0.99	0.10 ± 0.03	0.35
	ΔE	0.96	0.24 ± 0.31	0.21	0.97	0.05 ± 0.01	0.48
*O. basilicum-*D2	L*	0.93	0.14 ± 0.30	1.18	0.93	0.04 ± 0.01	0.06
	H*	0.92	0.17 ± 0.40	0.89	0.92	0.20 ± 0.03	0.94
	ΔE	0.96	0.01 ± 0.08	0.47	0.90	0.15 ± 0.02	0.56
*C. sinensis-*D2	L*	0.97	0.04 ± 0.17	2.08	0.96	0.03 ± 0.02	1.08
	H*	0.99	0.43 ± 0.19	0.03	0.91	0.06 ± 0.02	0.06
	ΔE	0.73	0.32 ± 4.04	0.16	0.92	0.09 ± 0.01	0.47
*O. vulgare-*D2	L*	0.95	0.12 ± 0.24	0.78	0.93	0.03 ± 0.04	1.78
	H*	0.96	0.08 ± 0.33	1.06	0.87	0.07 ± 0.02	0.05
	ΔE	0.94	0.41 ± 0.80	1.19	0.94	0.05 ± 0.02	0.16
*O. basilicum-*D4	L*	0.96	0.07 ± 0.23	0.01	0.94	0.03 ± 0.04	0.03
	H*	0.89	0.12 ± 0.56	0.06	0.90	0.05 ± 0.02	0.16
	ΔE	0.91	0.02 ± 0.36	0.53	0.88	0.04 ± 0.02	0.13
*C. sinensis-*D4	L*	0.93	0.14 ± 0.30	0.19	0.87	0.03 ± 0.05	0.06
	H*	0.92	0.11 ± 0.24	0.11	0.92	0.10 ± 0.02	0.17
	ΔE	0.97	0.43 ± 0.08	0.07	0.96	0.07 ± 0.03	0.09
*O. vulgare*-D4	L*	0.95	0.09 ± 0.23	0.14	0.93	0.02 ± 0.03	0.02
	H*	0.94	0.23 ± 0.42	0.09	0.94	0.05 ± 0.08	0.12
	ΔE	0.84	0.39 ± 2.13	0.11	0.97	0.11 ± 0.01	0.06
*O. basilicum-*D6	L*	0.94	0.23 ± 0.38	0.20	0.93	0.04 ± 0.03	0.51
	H*	0.97	0.14 ± 0.28	0.11	0.89	0.17 ± 0.07	0.15
	ΔE	0.87	0.31 ± 0.05	0.15	0.92	0.07 ± 0.03	0.05
*C. sinensis-*D6	L*	0.99	0.09 ± 0.11	0.13	0.98	0.14 ± 0.01	0.07
	H*	0.82	0.12 ± 0.41	0.09	0.94	0.05 ± 0.06	0.02
	ΔE	0.72	0.06 ± 0.09	0.19	0.96	0.12 ± 0.03	0.11
*O. vulgare*-D6	L*	0.93	0.23 ± 0.22	0.73	0.90	0.13 ± 0.06	0.06
	H*	0.72	0.36 ± 0.51	1.28	0.93	0.11 ± 0.05	0.09
	ΔE	0.94	0.05 ± 0.08	1.23	0.95	0.12 ± 0.03	0.12

R^2^, Coefficient of Determination; RMSE, Root Mean Square Error; K, Reaction rate; S, Spray; D2, Dip 2 min; D4, Dip 4 min; D6, Dip 6 min; L*, Lightness; H*, Hue angle; ΔE, Total color change.

Color parameters (L*a*b*) have the efficiency to demonstrate high degradation/loss with storage days, in the case of temperature-dependent it implies high activation energy (E_a_) during the storage period^35^. Most of the kinetic analysis study was determined using the first-order kinetic model and it was applied successfully to determine the quality changes in several food products^36^. The values of the rate constant specified to fluctuate for both parameters (L* and ΔE), which indicated color changes occur slowly in coated pear samples in the refrigerated condition (4 °C), therefore rate constant fluctuations showed that first-order kinetic model precisely accounted for the color degradation over storage period at 4 °C. The fractal dimension of the analyzed area along with kinetic analysis indicated greater complexity of L* values, i.e., a decrease in the lightness during browning kinetics over storage days^37^. Thus, the changes in color at each stage of fruit (maturation, ripening, and senescence) could affect the kinetics.

### Determination of shelf-life

Lightness and ΔE values obtained from image processing were selected as the color indicator for coated pear slices, signifying that these both parameters were suitable for the prediction of the shelf-life of *O. basilicum, O. vulgare,* and *C. sinensis* spray/dip-coated pear slices. To determine the expression that helps in the prediction of the shelf-life of coated pear slices concerning the changes in the lightness and total color change (ΔE), the predicted variable storage days were calculated concerning the storage temperature (4 °C). For this reason, the shelf-life was evaluated as the number of days until the quality/color changes showed a higher discoloration rate of the pears and thereby induced the symptoms of deterioration. Estimation of predicted shelf-life (PSL) with acceptable recommended values relied on the robustness of the kinetic models (Order model or Weibull model)^34^. For the determination of the shelf-life of PSL coated pear slices the first and zero-order kinetic model was used with storage conditions parameters (storage days) as per mentioned in Eqs. (7) and (8)^21,38^. The PSL of coated pear slices was determined by the threshold value of selected parameters. There were no notable change in the threshold values in terms of L* and ΔE, due to no observed variation.

The regression equations mentioned in Table 2 with R^2^ ranging from 0.86 to 0.98 were obtained by a linear fit of FOFC to determine ln(1−f) as the function of storage days at 4 °C. Using the Eq. (8) along with the regression equation from Table 2 helps in the prediction of shelf-life. Table 2 shows the PSL of coated pear slices using first-order kinetic and FOFC regression equations at storage temperature. The slight variation in L*, a*, b*, and ΔE demonstrated that storage days & temperature both implied a huge influence on the discoloration rate of the coated pear slices. The PSL for *O. basilicum* spray-coated pear slices at 4 °C were 28.85 and 26.11 days based on L* and ΔE, meanwhile for *O. vulgare* spray-coated pear slices at 4 °C was 27.22 and 25.86 based on L* and ΔE and *C. sinensis* spray coated pear slices at 4 °C was 28.63 and 27.95 based on L* and ΔE. Overall PSL using first order & FOFC kinetic modeling in spray-coated pear slices ranges in the sequence of *C. sinensis* > *O. basilicum* > *O. vulgare.*

**Table 2:** Shelf-life prediction of *Ocimum basilicum, Origanum vulgare* and *Camellia sinensis* extract spray/dip coated pear slices in relation to changes in color parameters at 4 °C

Edible coating	Color parameters	Regression equation	R^2^	PSL (days)
Control	L*	ln(1−f) = 0.16(1/T) + 5.27	0.97	16.00
	ΔE	ln(1−f) = 0.10(1/T) + 4.88	0.93	17.79
*O. basilicum*-S	L*	ln(1−f) = 0.05(1/T) + 7.91	0.89	28.85
	ΔE	ln(1−f) = 0.05(1/T) + 3.76	0.96	26.12
*C. sinensis*-S	L*	ln(1−f) = 0.11(1/T) + 2.72	0.92	28.63
	ΔE	ln(1−f) = 0.09(1/T) + 4.58	0.95	27.96
*O. vulgare*-S	L*	ln(1−f) = 1.17(1/T) + 8.11	0.96	27.23
	ΔE	ln(1−f) = 1.80(1/T) + 7.82	0.94	25.87
*O. basilicum-*D2	L*	ln(1−f) = 2.55(1/T) + 13.16	0.87	25.66
	ΔE	ln(1−f) = 2.26(1/T) + 16.72	0.90	24.74
*C. sinensis-*D2	L*	ln(1−f) = 0.03(1/T) + 8.94	0.97	26.22
	ΔE	ln(1−f) = 0.02(1/T) + 5.04	0.95	26.96
*O. vulgare-*D2	L*	ln(1−f) = 0.11(1/T) + 3.18	0.93	25.15
	ΔE	ln(1−f) = 0.14(1/T) + 7.93	0.92	23.75
*O. basilicum-*D4	L*	ln(1−f) = 0.12(1/T) + 4.08	0.96	23.01
	ΔE	ln(1−f) = 0.17(1/T) + 2.57	0.97	21.28
*C. sinensis-*D4	L*	ln(1−f) = 0.09(1/T) + 3.22	0.91	23.37
	ΔE	ln(1−f) = 0.09(1/T) + 4.72	0.93	24.80
*O. vulgare*-D4	L*	ln(1−f) = 0.14(1/T) + 2.40	0.95	22.62
	ΔE	ln(1−f) = 0.22(1/T) + 3.57	0.97	23.07
*O. basilicum-*D6	L*	ln(1−f) = 0.65(1/T) + 5.76	0.94	24.17
	ΔE	ln(1−f) = 0.51(1/T) + 3.22	0.91	21.13
*C. sinensis-*D6	L*	ln(1−f) = -0.08(1/T) + 3.86	0.88	23.04
	ΔE	ln(1−f) = -0.04(1/T) + 4.35	0.95	22.08
*O. vulgare*-D6	L*	ln(1−f) = -0.02(1/T) + 1.09	0.91	20.73
	ΔE	ln(1−f) = -0.08(1/T) + 3.86	0.95	21.71

R^2^, Coefficient of Determination; PSL, Predicted Shelf-life; S, Spray; D2, Dip 2 min; D4, Dip 4 min; D6, Dip 6 min; L*, Lightness; ΔE, Total color change.

Similarly, dip (2, 4, 6 min) coated pear slices showed predicted shelf life ranging from 26 ± 0.35 to 20 ± 0.12 days since the PSL obtained from *O. basilicum, O. vulgare,* and *C. sinensis* spray-coated pear slices was higher in comparison with the PSL obtained from the *O. basilicum, O. vulgare,* and *C. sinensis* dip coated pear slices. As PSL was calculated by two parameters (L* & ΔE), both the values for dip-coated samples were less due to the dark pigmentation formed in the initial days immediately after coating. It might be due to the formation of polymeric compounds on the surface^39^, yet the proven reason behind this discoloration is not identified. Among all the coatings, *C. sinensis* (0.1g/ml) spray-coated pear slices had the highest PSL based on L*and ΔE at 4 °C. Nevertheless, a FO & FOFC linear model best described the changes in PSL in terms of L* and ΔE, whereby achieving R^2^ greater than 0.86 for the spray/dip coated pear slices.

Thus, according to the results obtained, it can be concluded that the PSL model is considered to be an alternative approach that helps in the determination of shelf-life along with information that is useful to consumers regarding the storage days/condition of packed fruit supply. From this study, it is apparent that it is important to observe specific factors for PSL estimation, considering that the final information/data generates different possible outcomes.

## Methods

### Collection of raw materials

Three different herbs *Ocimum basilicum* (basil) leaves, *Origanum vulgare*** (**oregano) leaves, and *Camellia sinensis* (green tea) leaves in dried form and fresh pears (*Pyrus communis*) used in this experiment were purchased from the local market of Jalandhar, Punjab, India.

### Preparation of extract

Dried leaves of *Ocimum basilicum, Origanum vulgare, and Camellia sinensis* were grounded into a powder form using mortar and pestle. It was then extracted by hot aqueous extraction method (HAE)^40^, where powdered *Ocimum basilicum* leaves, *Origanum vulgare* leaves and *Camellia sinensis* leaves were dissolved in distilled water and heated in a water bath at 45 °C for 15 min. After cooling, the mixture was centrifuged at 10,000 g for 15 min. The supernatant was collected and used as an extract (0.1 g/ml).

### Preparation of pear slices

Purchased pears were washed using distilled water and pat-dried using a paper towel. The pat-dried pears were cut into pieces using a sterile knife having a thickness of 1 cm. The uniform thickness and shape of pears help in the selection of window frames for image analysis.

### Coating pear slices by herb extracts

Prepared herb (*Ocimum basilicum, Origanum vulgare, Camellia sinensis*) extracts were coated to all cut pears uniformly. Two different coating methods were chosen: (1) spraying and (2) dipping. To control film thickness and obtain thin films, the operation time for spray coating was fixed^41,42^. The fixed time for spray coating was set around 60 s and the time for dip coating was set as 2, 4, and 6 min for all three herb extracts. After coating, all of the pear slices were drained on stainless steel screens and then kept in plastic air-tight zip-lock pouches which were stored for 15 days at 4 °C for further experimental analysis.

### Titratable acidity

0.5 g of *O. basilicum, O. vulgare,* and *C. sinensis* extract-coated pear slices were crushed using mortar and pestle and further dissolved in distilled water. The prepared mixture was titrated against 0.1 N sodium hydroxide and 2–3 drops of phenolphthalein was used as an indicator^43^. The results were calculated according to the formula,

1 $${\text{Titratable}} \, \mathrm{ acidity }(\mathrm{\%})=\frac{{\text{Titre}} \, \mathrm{ value}\times {\text{Normality}}\times {\text{V}}1\times {\text{E}}\times 100}{{\text{V}}2\times {\text{W}}\times 1000}$$

where V_1_, Volume of mixture; V_2_, Volume of extract; W, weight of sample and E denotes the equivalent weight of NaOH.

### Quantification of discoloration rate

The surface discoloration rate quantification was carried with the help of image analysis technique^21^. The image captured was processed using a computer vision system (CVS) according to the protocol developed by Udomkun et al.^44^; Subhashree et al.^25^; Onwude et al.^45^ with slight modifications. Image analysis consists of three steps: Image acquisition, Image preprocessing, and Image processing.

### Image acquisition and cropping

To allow uniform illumination throughout the examination, a rectangular cardboard box was used of dimension 210*170*240 mm, and illumination was provided with a flashlight having 50 lumens. The inner wall of the rectangular cardboard box was covered with white chart paper, as this white background helps to get rid of reflections. To capture better quality images iPhone (operating system iOS16.3.1 having 458 Pixels Per Inch (ppi)) was used. The parameters notably ultra-wide aperture: f/2.4; digital zoom out: 10x; focus pixels: 100% (wide); max brightness: 1200 nits (HDR) were adjusted to suit the lighting conditions where the coated pear samples were exposed. The captured digital images were saved in JPEG format.

### Image preprocessing and processing

The median filter was used to preprocess the digital images of pear slices. Quality improvement and pre-smoothing of noisy digital images were done with the use of low pass filter. Further, processing of each pear slice images, color analysis, browning quantification and effective image texture analysis were determined using **“**Adobe Photoshop software (CS3 extended)—Version 10.0”. All pear slice images were processed using the “Adobe Photoshop software (CS3 extended)—Version 10.0” and measurements of RGB were done in 3 replications.

### Color features

The captured images contain three levels of primary colors: red, green, and blue commonly termed RGB colors. In general, L*a*b* values are used for the measurement of color values in food products, as this L*a*b* is considered very close to the color of human perception. At first, digital images of each coated pear slice (200*140 pixels) were chosen, and each image was cropped for extraction of a portion for the experimental analysis. Thus, the cropped digital images having RGB values must be first converted to L*a*b* using image analysis software. Color features notably lightness described as L* ranging from 0 to 100 where blackness is determined when L* = 0 and whiteness is determined when L* = 100 and parameter a* indicates color ranging from red to green where redness (positive) is determined when a* = 0 and parameter b* indicates color ranging from blue to yellow where yellowness (positive) determined when b* = 0. All these parameters determine the rectangular chromaticity coordinates (L*a*b*)^28^. The Lightness of the coated sample is expected to decrease over time. Along with these parameters, hue angle (H*) and total Color Difference (ΔE) were calculated using the equation as follows.

2 $$TCD(\mathrm{\Delta E})=\sqrt{{(\Delta L*)}^{2}+{(\Delta a*)}^{2}+{(\Delta b*)}^{2}}$$

3 $${\text{Hue}}\;{\text{angle}}\left( {H*} \right) = \, \tan^{ - 1} \left( {b*/a*} \right)$$

where ΔL* denotes lightness; Δa* denotes redness/greenness; Δb* denotes yellowness/blueness^17^.

#### Kinetic modeling consideration & shelf-life prediction

The experimental data obtained from L*a*b* values were fitted using different models to obtain valuable descriptions of the changes produced in the color attributes of the coated pear slices for 15 days of storage. The models used to fit experimental data were zero-order, first-order, and first-order fractional conversion (FOFC)^46^. The zero-order and first-order chemical kinetic are defined in the following equation.

4 $${\text{First - order chemical kinetics }}\left( {{\text{n}} = {1}} \right):{\text{ C}}_{{\text{t}}} /{\text{C}}_{0} = {\text{ exp }}\left( { - {\text{kt}}} \right)$$

5 $${\text{Zero - order chemical kinetics }}\left( {{\text{n}} = {2}} \right):{\text{ C}}_{{\text{t}}} - {\text{ C}}_{0} = \, - {\text{kt}}$$

where C_0_, initial value of quality factor; C_t_, value of quality factor with time (t); k, reaction rate and t, storage time.

First-order fractional conversion is defined as the conversion of reactant fraction to a specific product at a given time. In place of concentration, this fraction conversion is used as a convenient variable^47^, thus, this technique is used usually at the end of kinetic examination when a traceable amount of reactant remains after a specific reaction time and it helps in kinetic data reduction. FOFC can be determined by *f* as a quality index and the following equation is derived concerning the reaction rate at a constant temperature.

6 $$f = \left( {{\text{C}}_{0} - {\text{C}}_{{\text{t}}} } \right)/\left( {{\text{C}}_{0} - {\text{C}}_{\infty } } \right)$$

where C_0_, the initial quality factor of the fruit, C_t_, quality factor after a specific time t, and C∞, final quality factor at non-zero equilibrium value. All these fitting was done with the help of OriginPro 2023 (The ultimate software for graphing and analysis). Predicted shelf life (PSL) at the actual storage condition (15 days) can be calculated using the below equation^38^.

7 $${\text{If}}\;{\text{n}} = 0\left( {\text{Zero - order}} \right),{\text{PSL}} = \left( {{\text{C}}_{{\text{t}}} {-}{\text{C}}_{0} } \right)/ \pm {\text{k}}$$

8 $${\text{If}}\;{\text{n}} = {1}\left( {\text{First - order}} \right),{\text{PSL}} = \left( {{\text{ln}}\;{\text{C}}_{{\text{t}}} {-}{\text{ln C}}_{0} } \right)/ \pm {\text{k}}$$

#### Statistical analysis

The experiments were conducted in triplicates and a completely randomized design. Excel 2016 and IBM SPSS statistic software were used to analyze experimental data. The statistically significant differences among *O. basilicum, O. vulgare, and C. sinensis* sprayed/dip-coated pear slices were determined by analysis of variance (ANOVA) and Turkey’s range at a confidence interval of *p* < 0.05. OriginPro 2023 (The ultimate software for graphing and analysis) was used to interpret graphical data.

## Conclusion

To evaluate the effectiveness of edible *Ocimum basilicum, Origanum vulgare,* and *Camellia sinensis* extract coating applied on pear slices, color parameters (L*, a*, b*, H*, ΔE) of coated pears are measured as indicators. Color is a critical parameter in kinetic-based PSL that determines the shelf-life of the coated pears, and the shelf-life predicted was accurate by a similar type of product. This study confirmed that coating herb extracts to pear slices in combination with refrigerated storage helps to decrease the discoloration rate for a certain period of days. Among three herbal extracts, *C. sinensis* spray-coated pear slices showed a lower discoloration rate and the highest PSL (28.63 and 27.95 based on parameters L* and ΔE). The overall discoloration rate was minimum observed in the order of *C. sinensis* > *O. basilicum* > *O. vulgare* extract spray-coated pear slices. Overall examination throughout this study using image analysis showed better accurate results in comparison with similar analysis. As this kind of coating is confirmed to decrease the discoloration/browning rate, it can be applied on a large scale, which gains profit for food processors with a longer shelf-life and improved product safety in comparison with chemically coated fruits.

## Data Availability

All the data generated and referred to during this study are mentioned in this article.
