# Author Correction: Image analysis-based discoloration rate quantification and kinetic modeling for shelf-life prediction in herb-coated pear slices

**DOI:** 10.1038/s41598-024-53545-8

**Published:** 2024-02-06

**Authors:** Sathya R., Prasad Rasane, Aishvina Singh, Jyoti Singh, Sawinder Kaur, Vikas Nanda, Jaspreet Kaur, Mahendra Gunjal, Vishesh Bhadariya, Sezai Ercisli, Riaz Ullah, Essam A. Ali

**Affiliations:** 1 Department of Food Technology and Nutrition, School of Agriculture, Lovely Professional University, Phagwara, Punjab 144411 India; 2 Sant Longowal Institute of Engineering and Technology, Sangrur, Punjab 148106 India; 3 School of Chemical Engineering, Oklahoma State University, Stillwater, OK-74078 USA; 4 Department of Horticulture, Faculty of Agriculture, Ataturk University, 25240 Erzurum, Turkey; 5 HGF Agro, ATA Teknokent, TR-25240 Erzurum, Turkey; 6 Department of Pharmacognosy, College of Pharmacy, King Saud University, Riyadh, Saudi Arabia; 7 Department of Pharmaceutical Chemistry, College of Pharmacy, King Saud University, Riyadh, Saudi Arabia

Correction to: *Scientific Reports* 10.1038/s41598-024-51840-y, published online 18 January 2024

The Acknowledgements section in the original version of this Article was omitted. The Acknowledgements section now reads:

“Authors wish to thank Researchers Supporting Project number (RSP2024R110) at King Saud University Riyadh Saudi Arabia for financial support.”

The original Article has been corrected.

